# Parotid Gland Metastases of Cutaneous Squamous Cell Carcinoma of the Head: Occult Metastases Occurrence and Their Late Manifestation

**DOI:** 10.1155/2024/5525741

**Published:** 2024-02-19

**Authors:** Zuzana Horakova, Ivo Starek, Jana Zapletalova, Richard Salzman

**Affiliations:** ^1^Department of Otorhinolaryngology and Head and Neck Surgery, University Hospital Olomouc, Faculty of Medicine and Dentistry, Palacky University Olomouc, Zdravotniku 248/7, Olomouc 77900, Czech Republic; ^2^Department of Medical Biophysics, Faculty of Medicine and Dentistry, Palacky University, Hnevotinska 3 77515, Olomouc, Czech Republic

## Abstract

Cutaneous squamous cell carcinomas (cSCC) are malignant tumours with excellent prognosis unless nodal metastases develop. The aim of our study is to determine the prognostic significance of the clinical stage of parotid gland metastases and the incidence of occult cervical lymph node involvement in cSCC of the head. Our retrospective analysis includes 39 patients with cSCC parotid gland metastases, 15 of whom had concurrent cervical node involvement. In 32 patients, the lymph nodes manifested at stage N3b. A total of 26 patients were treated with parotidectomy, 9 patients received radiotherapy alone, and 4 received symptomatic therapy. The surgical treatment included either total conservative (21 cases) or superficial parotidectomy (5 cases) and neck dissection (therapeutic neck dissections in 11 cases and elective in 14 cases). In all cases, surgery was performed with sufficient tumour-free resection margins. Adjuvant radiotherapy was administered postoperatively in 16 patients. Occult metastases were present in 21% of cases after an elective neck dissection, but not in any case in the deep lobe of the parotid gland. The five-year overall survival and recurrence-free interval were 52% and 55%, respectively. Patients with the cN3b stage and G3 histological grade tend to have a worse prognosis, but not at a statistically significant level. The prognosis was not worse in patients with concurrent parotid and cervical metastases compared to those with metastases limited to the parotid gland only. The addition of adjuvant irradiation, in comparison to a single modality surgical treatment, was the only statistically significant prognostic factor that reduced the risk of death from this diagnosis (*p*=0.013). The extent of parotidectomy (partial vs. total) had no impact on either the risk of recurrence or patient prognosis. The combination of surgery with irradiation provides the best results and should be applied to all patients who tolerate the treatment. A partial superficial parotidectomy should be sufficient, with a minimum risk of occult metastasis in the deep lobe. Conversely, the relatively high incidence of occult neck metastases indicates that patients could likely benefit from elective neck dissection.

## 1. Introduction

Squamous cell (SCC) salivary gland cancer accounts for only about 5% [1, 2] of the currently identified salivary histopathological units [3]. The vast majority of all salivary SCC are located in the parotid gland. From all parotid SCC, only 10–20% account for primary tumours originating from the parenchyma of the gland. Typically, over 80% of tumours are of metastatic origin, characterized by nodal, intraparotid or paraparotid, metastases of primary SCC. These primaries are located mainly on the skin of the face and anterior scalp [4]. The primary tumour can also, though much less frequently, originate from SCC of a mucous membrane of the upper aerodigestive tract, especially the nasopharynx [5] and oropharynx [4]. Exceptionally, kidney, lung, GIT, prostate, or breast tumours can also metastasize to the parotid lymph nodes [6]. The reported incidence of cutaneous SCC (cSCC) lymph node metastases at all tumour sites is very low, 2–5% [1, 2], while SCC located on skin of head and neck are considered high-risk, with an incidence of metastases of 6–8% [7, 8]. Additionally, some clinical and histological tumour parameters further increase the risk of metastases: tumour size and tumour invasion depth, tumour recurrence, site of prior radiotherapy, low grade of differentiation, perineural, lymphatic, and vascular involvement, and immunosuppression of a patient [3]. However, the incidence of occult metastasis even in the high-risk head and neck cSCC does not exceed 20%, which is a generally accepted limit indicating an elective treatment of regional lymph nodes [9].

The prognosis of cSCC after adequate surgical treatment is very good. The 5-year overall survival (OS) exceeds 90% [10–12]. However, the prognosis of patients with cSCC parotid metastases is relatively poor with the five-year OS rarely exceeding 50% [11–17]. This may be due to the fact that, in addition to their high biological aggressiveness, most patients are not always adequately followed up after radical resection of primary skin cancers, which can lead to delays in detection and therapy of parotid metastases. Another negative factor appears to be the fact that despite the known predictive, NCCN [3] defined factors of lymphogenic manifestation of cSCC of the head, preventive cervical dissection or parotidectomy are currently not indicated. For patients with clinically present parotid metastases, surgical management and adjuvant radiotherapy, including cervical lymph nodes dissection, are indicated, even in cN0 necks [18, 19]. Neck dissection in these cases has an elective character and is carried out selectively at levels consistent with the location of the primary tumour, e.g., neck level II, III, and possibly also level V for skin tumours of posterior scalp. The microscopic positivity of cervical nodes in cN0 patients with parotid lymph node involvement is usually above 30% [5, 11, 20–22]. However, according to some authors, it is below 20% [23, 24], and consequently, these authors consider elective neck dissections to be an unnecessary burden for a patient.

In this paper, we assess the prognostic significance of parotid and cervical lymph node metastases in cSCC of the head, as well as the incidence of hidden cervical lymph node involvement in patients with parotid metastases.

## 2. Materials and Methods

### 2.1. Diagnostic Methods

A retrospective study analysed medical data on patients treated with SCC of a parotid gland over a 12-year period (2008 to 2020) in one tertial ENT department.

The Declaration of Helsinki was followed and approval of the Institutional Ethical Board was received.

The staging of primary tumours and lymph nodes was conducted according to the current TNM classification, [25] and the extent of parotid metastases was also determined according to O'Brien [11]. This was done for all patients before the start of treatment. The skin tumour was evaluated by a dermatologist, and cervical and parotid nodes were examined both clinically and with relevant imaging methods (US, CT, MRI, PET/CT). Simultaneously, duplex malignancy was ruled out by this diagnostic procedure. The histopathological diagnosis of squamous cell carcinoma of the skin tumour was confirmed postoperatively by examining surgical specimens, or after the excision of the biopsy sample in inoperable tumours. Lymph node status was assessed in clinically evident nodal metastases using aspiration cytology (only in those cases where the diagnosis was later confirmed by histology after surgical treatment) or core needle biopsy.

Histologically, the diagnosis of cSCC primary tumour and regional metastases was primarily based on the basic morphology in hematoxylin-eosin staining, which revealed the presence of keratinization also confirmed immunohistochemically by the presence of typical markers, particularly p40, p63, CK5/6. HPV status was determined immunohistochemically by p16 expression to exclude the oropharyngeal origin of metastasis. The grading (the degree of differentiation) of the tumour was estimated.

Information on previously treated cSCC and the interval between the removal of the primary skin tumour and the clinical manifestation of its lymph node metastases was identified by asking each patient directly and confirmed at the corresponding medical institute where the primary skin tumour was excised. Clinical data were regularly updated during the follow-up.

### 2.2. Set of Patients

Forty-seven patients were enrolled in the retrospectively. Epidemiological and clinical data on patients are presented in Table 1.

Eight out of 47 patients with histologically diagnosed parotid SCC were excluded as they met both clinical and histopathological criteria for the primary lesion [26].

(Any primary tumour located extraglandulary was excluded by patient history, PET/CT scan, endoscopy of the upper aerodigestive tract, and dermatologic examination.)

Parotid metastasis from other than cutaneous primary tumour was not diagnosed in any patient; all parotid SCC were p16 negative.

The remaining 39 cases were primary cSCC metastases, of which metastases were detected in either in parotid nodes exclusively in 24 patients or simultaneously in parotid and neck lymph nodes in 15 patients. This population included 32 men and 7 women, ranging in age from 41 to 95 years (76 on average). The study did not include 3 cSCC of the head patients whose primary tumour metastasized exclusively to cervical lymph nodes.

### 2.3. Statistical Analyses

The effect of clinical (cN) stage, histopathological grading (G), localization of lymph node metastases, and treatment of the cSCC on patient prognosis was analysed.

The predictive importance of each parameter was determined using Fisher's exact test. Overall survival (OS) and disease-free interval (DFI) were evaluated using the Kaplan–Meier analysis and compared using the log rank test.

**Figure 1:**
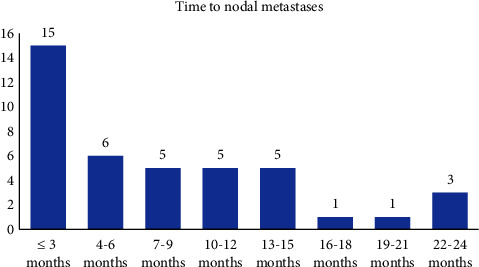
Histogram showing time period between primary skin tumour resection and parotid metastasis manifestation.

The interval between the removal of the primary skin tumour and the clinical manifestation of its lymph node metastases is shown in Figure 1.

IBM SPSS Statistics Version 23 (Armonk, NY: IBM Corp.) statistical software was used to analyse the data. All tests were considered to be significant at a level of 0.05.

The significance of risk factors for metastasis was assessed using the Cox proportional hazard model.

## 3. Results

### 3.1. Tumour Primary Location and Parotid and Cervical Lymph Node Involvement

**Figure 2:**
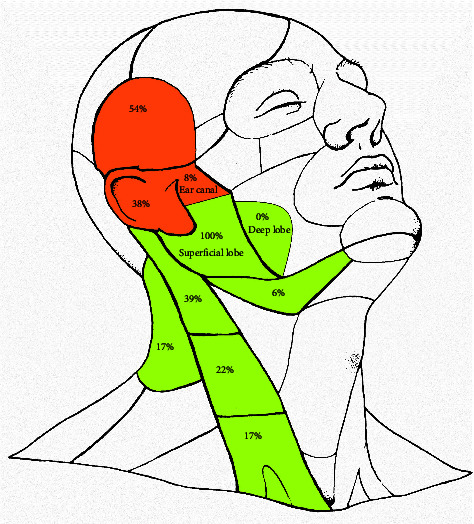
Distribution of primary skin tumours and their regional metastases. Localization and frequency of primary cSCC in 39 patients in temporal, auricular, and external ear canal location (orange areas) and their parotid (superficial and deep lobe) and cervical lymph node metastases (green areas).

In 21 patients, the cutaneous primary tumours were located in the frontotemporal region, in 15 on auricula and in 3 in the cartilaginous external ear canal (Figure 2). Five (13%) of the 39 patients were classified as cN + at the time of primary tumour resection. Thirty-four (87%) cases were classified as cN0 at the time of primary skin tumour resection. Lymph node involvement clinically manifested in 324 (average 8) months after treatment of primary tumour, of which 9 at the time of its local recurrence (Figure 1). The history of nodal metastases ranged from 1 to 34 (mean 7) weeks. Of the total population, 2 patients were diagnosed with cN1, 5 with cN2b, and 32 with cN3b. All 32 cN3b patients had histological confirmation of extracapsular extension (ECE). Of the 24 patients with exclusively parotid lymph node involvement, 19 were diagnosed with stage cN3b, 2 with stage cN1, and 3 with stage cN2b.

In 5 out of 15 patients with simultaneous metastases in both parotid and cervical lymph nodes, only one level (level II in all cases) was involved, in other 10 patients metastases were detected in two levels (1x I + IV, 1x II + V, 3x II + III) or four or more (4x II-V and 1x I–V) levels. (Figure 2)

Parotid metastases showed a degree of G1 differentiation in 4 patients, 12 patients G2, and other 16 patients G3. The histopathological grading could not be determined in 7 patients who were treated with radiotherapy alone and lymph node metastases were diagnosed with a core-needle biopsy.

### 3.2. Therapy

The primary skin tumour was removed surgically in all but one patient who received radiotherapy. Of the 9 patients with locoregional recurrence of the skin tumour, 7 were treated with a combination of surgery and adjuvant radiotherapy and 2 with radiation alone.

In 22 patients with the cN3b stage, a conservative total parotidectomy was performed and 4 patients with cN1-2 underwent a superficial parotidectomy. Negative resection margin (R0) was reached in all total and superficial parotidectomies performed. No patient presented a metastatic involvement of the deep lobe of parotid gland after total parotidectomy.

**Figure 3:**
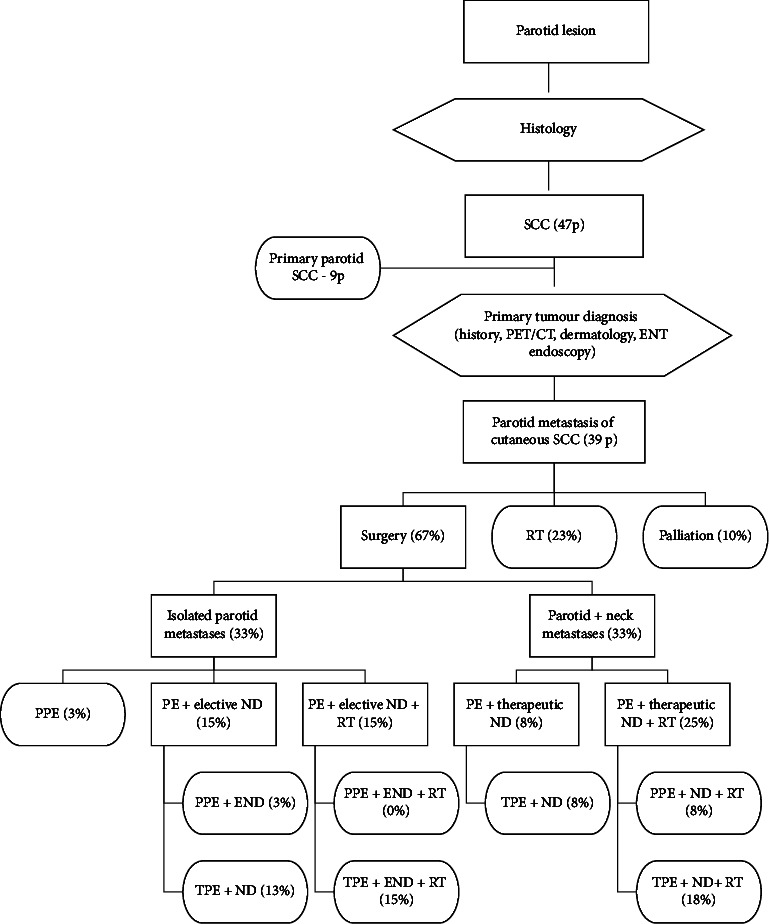
Flow diagram showing cohort of patients with parotid SCC, diagnostic procedure, and distribution of applied treatment. SCC: squamous cell carcinoma; p: patient; PET/CT: positron emission tomography/computed tomography; ENT: otorhinolaryngology; RT: radiation therapy; PE: parotidectomy; PPE: partial parotidectomy; TPE: total parotidectomy; ND: neck dissection, END: elective neck dissection; CHRT: chemoradiotherapy; and II, III, V: levels of the neck nodes.

In 9 patients, only a palliative radiotherapy was administered and symptomatic therapy in other 4 patients. These patients could not undergo a radical treatment due to the extent of the local tumour or their general condition. (Figure 3)

Of the 24 patients with exclusive parotid gland involvement, 15 had undergone surgical treatment, with 14 undergoing elective neck dissection in addition to parotidectomy. Of these 14 patients, occult cervical lymph node metastases were detected in 3 cases (21%). Nine of them underwent adjuvant radiotherapy.

Eleven out of 15 patients with concurrent parotid and cervical lymph node involvement were treated with a curative neck dissection in addition to parotidectomy, followed by adjuvant radiotherapy in 10 patients (1 patient refused radiotherapy). In the others, only a palliative or symptomatic therapy was administered.

### 3.3. Prognosis

**Figure 4:**
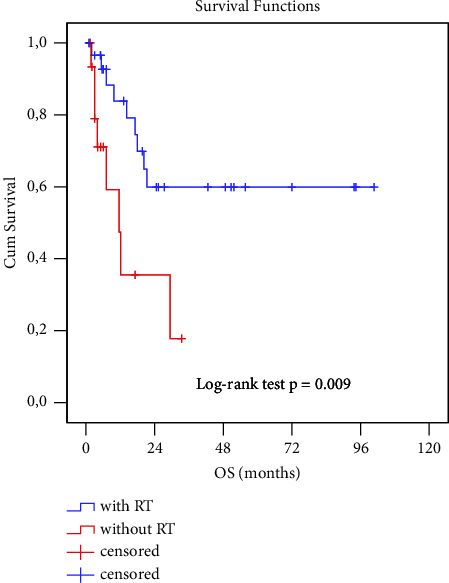
Kaplan–Meier analysis of OS comparing patients with combined (surgery + irradiation) and single modal (surgery) treatment.

For the whole group of 39 patients, the mean survival time was estimated to be 59.3 months (std. error 9.0; 95% CI 41.6–71.0). Twenty-three of the 26 curatively treated patients achieved complete remission. Median time to relapse, (disease-free interval (DFI)) was 64 months (CI 95% 45–83); DFI mean 0.59 (std. error 10.5; 95% CI 38.9–80.1), median 94.4 (std. error 51.9, 95% CI 0–196.1). A 5-year OS and DFI was 0.52 (65% CI 0.31–0.73) and DFI 0.55 (65% CI 0.30–0.80) (Figure 4). Seven patients experienced a lymph node relapse (6 times in parotid and 1 time in cervical lymph nodes) and one patient developed a distant relapse of the disease. The follow-up interval was 2–125 (mean 31) months.

### 3.4. Treatment Modality

The impact of a treatment modality was evaluated by comparing patients treated by combination of surgery with postoperative irradiation versus patients treated with surgery only.

The median and mean survival time estimates were 15.7 months (standard error 3.8; 95% CI 8.2–23.25), median 11.6 months (standard error 3.4) after single modality treatment, and 65.7 months (standard error 9.2; 95% CI 47.8–83.7) after a combination of surgery and radiotherapy.

**Figure 5:**
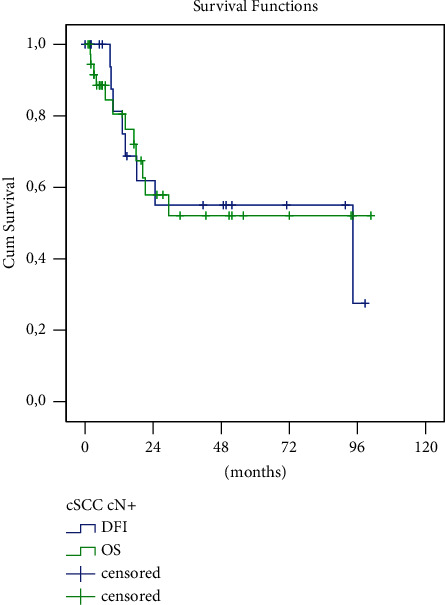
Kaplan–Meier analysis of overall survival (OS) of the whole set of patients and time without disease recurrence (DFI).

This difference was highly significant (log-rank test *p*=0.009), with postoperative therapy reducing the risk of death by 0.293-fold (*p*=0.013); see Kaplan–Meier analysis (Figure 5, Table 2). There was no decline in OS of the second group after 5 years. This parameter could not be determined for the surgery only group. However, when comparing the probabilities of median survival, a significant difference was found (OS was 0.596 vs 0.178 (CI 0.392–0.805; 0–0.468).

### 3.5. Distribution of Nodal Metastases

There was a positive trend suggesting a better survival in patients whose parotid lymph nodes were classified as cN1-2 (according to O'Brien P1-2) compared to cN3 (P3 according to O'Brien) patients (*p*=0.173). However, this trend was not observed when assessing the condition of all affected lymph nodes, including both parotid and cervical lymph nodes, according to the latest TNM classification. The result is likely altered by the nonhomogeneous distribution of the *N* values of the entire group, with most (32 out of 39) parotid gland metastases already at the most advanced stage of extracapsular spread and only a small proportion (7) at the lower stages.

Parotid metastases with G1-2 differentiation did not show significantly worse OS or DFI compared to those with G3 differentiation (*p*=0.364, respectively, *p*=0.284, log rank test).

**Figure 6:**
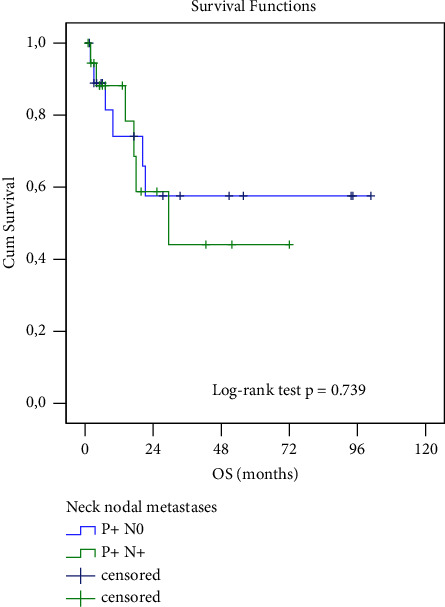
Kaplan–Meier analysis of overall survival comparing patients with only metastatic parotid gland disease (P + N0) and concurrent parotid and cervical lymph node disease (P + N+).

When comparing OS or DFI between the subgroup of patients with parotid lymph node involvement only and the subgroup with concurrent parotid and cervical lymph node involvement, the difference was not statistically significant (58% vs. 42%, resp. 62% vs. 48%, log rank test, both *p* > 0.05). (Figure 6, Table 2)

### 3.6. Superficial vs. Total Parotidectomy

A total of 83% of patients who underwent superficial parotidectomy and 50% of those who underwent total parotidectomy survived for 5 years after the end of treatment (CI 0.535–1.0 and 0.248–1.0, respectively). The five-year disease-free interval (DFI) after treatment was 80% for patients who underwent superficial parotidectomy and 53% for patients who underwent total parotidectomy (CI 0.449–1.0 and 0.250–0.805, respectively). The difference between the two surgical procedures was not statistically significant for any of the listed prognostic parameters, but both showed a more favourable prognosis for the less extensive procedure (*p*=0.264 and 0.468, respectively, log rank test).

### 3.7. Parotid Deep Lobe Metastases

In the histopathological examination of the specimen after total parotidectomy, metastatic involvement of the deep lobe of parotid gland (0%) was not detected in any of the cases.

All analysed parameters and their significance to patients' prognosis (OS, DFI) are summarized in Table 2.

## 4. Discussion

Our study demonstrated a very poor prognosis of cutaneous squamous cell carcinomas (cSCCs) with metastases to the parotid gland, with a 3-year survival rate of only 0.48. Numerous other studies [15–17, 23, 27–34] confirmed the severity of the prognosis, with 5-year survival (OS) ranging from 50-65% and disease-free interval (DFI) of 55–75%. Similarly, only one-third of patients survived 5 years in Lee's study [17]. The explanation is that the majority of cases (as in our work) are diagnosed late at the most advanced clinical stage (N3b), which, according to Ch'ng [15] and Audet [24], represents the only independent negative prognostic factor.

The late diagnosis was undoubtedly due to the fact that very few patients had their parotid lymph nodes regularly checked by clinical examination or relevant imaging modality. Eight cases (17%) of our entire set of 47 parotid SCCs met the pathological and clinical criteria of primary SCC of the parotid gland. Primary SCC of the parotid gland originates in the metaplasia of the ductal epithelium of the salivary gland. This diagnosis is confirmed if the tumour directly affects the tissue of the salivary gland without communication with the skin and at the same time presence of mucin or residual lymph node structure is excluded. In addition, any previous or current especially cutaneous malignancy must be ruled out in each patient [26]. The very low incidence of the latter histopathological unit was also reported by Franzen [1] and Aboziada [13]. Some authors even deny its existence [5, 34].

In the study, patients with well and moderately differentiated parotid metastases tend to have a better prognosis than those with low-grade ones. This finding correlates with the work of Phister [28], who found that the difference between well and poorly differentiated tumours reached statistical significance.

Other prognostic factors for SCC parotid metastases such as a facial nerve palsy [17, 24, 30], extraglandular spread [13, 14], and black race origin [28] were identified. However, in our cohort, these factors have not been investigated for their extremely low frequency. Our patients' age did not seem to be prognostically significant in contrast to other authors [14, 17, 28].

In our study, on average, parotid gland metastases clinically manifested at 8 months after treatment after their primary cutaneous tumour resection with a maximum of 24 months. Our data correlate with findings of other authors [27, 31, 32, 34], according to which this late manifestation ranges between 4 and 13 months. The longest interval in these studies did not exceed 3 years. A standard follow-up period for cSCCs of 5 years seems sufficiently justified and should include not only a local skin examination but also the parotid and cervical lymph nodes imaging.

Another argument for a close follow-up is the rapid growth dynamics of parotid metastases. The vast majority (33 of 39 patients, 85%) of patients were diagnosed with metastases at the highest clinical stage (N3b), with an average of only a 7-week history of resistance in the parotid gland (median 4 weeks). In all cases, extracapsular extension (ECE) was present, which is an important negative prognostic parameter. Similarly, higher incidence of ECE in parotid metastases compared to cervical lymph node metastases (up to 40%) [8] was also published by Yesensky (85%) [35] and Khurana (70%) [27]. The extracapsular spread of metastasis outside the lymph node capsule (ECE) is considered to be a negative prognostic biological factor which is also taken into consideration in the TNM classification. However, the biological reason for early presence of ECE in intraparotid lymph nodes is not yet elucidated [35, 36].

Our study showed a significantly better prognosis in patients who underwent parotidectomy with either adjuvant radiotherapy or chemoradiotherapy (5-year OS and DFI, both 60%) compared to patients who underwent only surgery without irradiation (5-year OS 17% and DFI cannot be determined). Similar results were reported by other authors [14, 16, 21, 24, 30, 33].

The combined therapy of surgery and adjuvant RT was the preferred treatment option, suggested for all patients who could tolerate the treatment [33]. Similarly, in our study, surgery with irradiation offered the best survival results, significantly better than any other single modality treatment. Chen published database results (SEER) from 1988–2009. He identified 2104 adult patients with parotid SCC. Comparing surgical and nonsurgical treatment, surgery was associated with an improved 5-year DSS (44.4% vs 71.0%; *p* < 0.001), whereas radiation alone was similar to no treatment (47.0% vs 41.6%; *p*=0.28). [14].

Several independent studies confirmed that the best prognosis was offered by the combination of surgery and adjuvant RT. It provided a statistically significant improvement in locoregional control over preoperative RT or RT alone (83% vs. 59% and 47%, respectively) [16].

Taylor presented that the ultimate rates of disease control in the parotid area after combined surgery and irradiation were excellent (89%), in comparison to surgery alone (63%) and irradiation alone (46%) [30]. Audet published very similar results. The overall recurrence rate was 29%. DSS was significantly better in patients treated with surgery + RT compared to irradiation alone (*p* < 0.05) [24].

For parotid gland carcinomas of advanced clinical stage and/or high biological risk, including cSCC metastases, removal of the whole gland is recommended [5, 23, 28, 37–44]. In our cohort of patients, there was no difference in OS and DFI between patients after total and superficial parotidectomy. The conclusions of retrospective analyses [37, 45, 46] also show no difference in survival between patients with parotid metastases of cSCCs after total and superficial parotidectomy. Superficial parotidectomy was even more prevalent (60%) in Kampel's study [37], while total parotidectomy was only performed in the presence of an apparent deep lobe macroscopic involvement. We have not found any prospective studies on this issue in the literature.

In our study group, majority of patients underwent total parotidectomy. Occult metastases in the deep lobe of parotid gland have not been confirmed in any patient.

Partial superficial parotidectomy was performed only in those patients whose finding on parotid lymph nodes in the superficial lobe was assessed as N1-2. In all of these cases, negative resection margins (R0) were reached. In none a parotid recurrence has developed.

This result is supported by the current NCCN [3] recommendations to perform only superficial parotidectomy even in metastatic involvement of the parotid gland [37].

In contrast, the Dur's [18] study indicates a 28% incidence of deep lobe metastases (with 5% isolated in the deep lobe only) supporting the indication for more extensive resection. This is also supported by the Thom's study [19], in which a set of 42 patients with metastatic superficial lobe involvement after total parotidectomy with neck dissection were detected with occult metastases in the deep lobe in 26% and in the cervical lymph nodes in 31% (in most cases, there were occult metastases detected simultaneously at both sites). Thom's treatment achieved a very good locoregional disease control of 93% after 5 years; however, he could not compare his data with a control group after superficial parotidectomy only.

The questions remain whether adjuvant radiotherapy in this indication would be sufficient to cure any deep lobe micrometastases [18, 37] and if superficial parotidectomy can be considered a sufficiently radical procedure.

Our work did not confirm a worse prognosis for patients with concurrent cervical lymph node involvement when compared to those with parotid metastases only. This finding is, thus, in agreement with the results of other studies [13–15, 17]. However, several authors [10, 15, 16] report significantly better prognosis in patients without concurrent cervical lymph node involvement. In our study group, we only proved a trend toward a worse prognosis for patients with cN3b stage of parotid lymph nodes, regardless of the stage of cervical lymph node involvement. The outcome may have been influenced by the inhomogeneity of the set of patients dominated by the most advanced N stage. However, the results of the O'Brien's study [11] suggested that the condition of the parotid lymph nodes (regardless of cervical lymph nodes) is an independent prognostic factor. The author, therefore, has concluded that the condition of parotid nodes should be evaluated as a separate prognostic parameter. He named it P stage in TPNM classification. However, the work by Forest [47] disagreed with the conclusions of O'Brien [11], and the current TNM classification [25] does not respect a separate evaluation of parotid lymph nodes.

In our study, 14 patients underwent elective neck dissection as part of their surgical treatment, and in 3 of them (21%) histology revealed occult metastases. The relatively high incidence of occult neck metastases indicates that this group of patients could likely benefit from elective neck dissection, and conversely, the removal of deep lobe of the parotid gland might not be indicated.

According to other literary sources, their incidence in the secondary SCC of parotid gland ranges between 16-44%, and their authors, therefore, recommend elective neck dissection in these cases [5, 23, 24, 41, 42, 48]. The positive impact of this procedure on the prognosis was confirmed by Phisterer [28] in an extensive retrospective analysis, with survival rates of 78.3% for patients after both elective neck dissections and parotidectomy, compared to 51.1% where only parotidectomy was performed.

In patients with parotid lymph node metastases, the selection of neck lymphatic regions included in elective neck dissection should be determined by the localisation of the primary skin tumour [29, 32, 48]. When skin of the face is affected, it is recommended to perform a neck dissection in levels II and III. For auricular and fronto-temporo-parietal carcinomas, this procedure is recommended in level Va in addition to levels II-III. However, the reported incidence of occult metastases does not exceed 4% in level VA [49]. This recommendation is supported by our study which included only primary skin cancers of the two latter sites and showed a higher than 20% risk of metastases in levels II (39%) and III (22%). In our study, only a single case (7%) from all our 14 elective dissections (in levels II, III and VA) showed metastases at the VA level.

In the case of clinical N+ neck, a comprehensive neck dissection in areas I–V should be performed [10, 48].

## 5. Conclusion

Lymph node metastases of the cSCC of the head were in 60% in limited at initial diagnosis to parotid region only. A vast majority (85%) were detected in the most advanced clinical stage (N3b), already with extracapsular spread. 67% were treated with a combination of surgery and adjuvant irradiation, and in 40% of cases, adjuvant radiotherapy was added. The growth rate of parotid metastases is very fast. We have observed a tendency for worse survival in patients with extensive lymph node involvement, but simultaneous involvement of the cervical lymph nodes does not worsen the disease.

In our patient cohort, we did not observe a clear advantage of total parotidectomy over superficial parotidectomy. Furthermore, no metastases were identified in the deep lobe of the parotid gland specimen resected during total parotidectomy. This suggests that superficial parotidectomy is a sufficiently radical surgical treatment for patients with tumours macroscopically limited to the superficial parotid lobe.

On the other hand, we found occult metastases in the neck lymph nodes in 21% of patients with isolated parotid gland involvement. This finding indicates that elective neck dissections should be performed in these cases.

The only statistically significant parameter that had a positive effect on the otherwise very poor survival of cSCCs with nodal metastases was the addition of adjuvant therapy to the initial surgical treatment. Therefore, we recommend that all patients who can tolerate combined therapy undergo it to achieve the best prognosis.

## Figures and Tables

**Table 1:** Epidemiological and clinical data of patients, staging of primary tumours and lymph nodes according to current TNM classification (TNM), and extent of parotic metastases according to (O'Brien).

Number of patients		39
Sex	Males/females	32/7
Age	Min-max; mean	41–95; 79
Primary skin tumour T	T 1 : 2 : 3 : 4	13 : 9 : 13 : 4
Regional value of cN	cN 1 : 2 : 3	2 : 5 : 32
*P* value in patiens with exclusively parotid metastases	cP 1 : 2 : 3	2 : 3 : 19
*P* value in patiens with parotid and cervical metastases	cP 1 : 2 : 3	0 : 2 : 13
*N* value for cervical lymph nodes	N 0 : 1 : 2 : 3	24 : 1 : 8 : 6
Stage	I : II : III : IV	0 : 0 : 2 : 37
Grade^*∗*^	G I : II : III	4 : 12 : 16

^*∗*^in 7 patients histological grading was not assessed.

**Table 2:** Significance of analysed parameters in relation to survival and time to recurrence (OS, DFI).

		OS (log rank test)	DFI (log rank test)
Extent of intraparotic metastases	P1 + 2 vs P3	NS	NS
Extent of neck nodal metastases	N0 vs cN+	NS	NS
Extent of regional nodal metastases	N1 + 2 vs N3	NS	NS
Grade of differentiation	G1 + 2 vs G3	NS	NS
Type of parotidectomy	Partial superficial vs total parotidectomy	NS	NS
Adjuvant radiotherapy	RT+ vs RT−	0.009	NP^*∗*^

NS—not significant, NT—not tested. ^*∗*^DFI was not tested due to low number of patients without RT.

## Data Availability

The data used to support the findings of this study are available from both corresponding and first authors.
